# A potential global surveillance tool for effective, low-cost sampling of invasive *Aedes* mosquito eggs from tyres using adhesive tape

**DOI:** 10.1186/s13071-020-3939-0

**Published:** 2020-02-19

**Authors:** Thom Dallimore, David Goodson, Sven Batke, Clare Strode

**Affiliations:** Department of Biology, Edge Hill University, St. Helens Road, Ormskirk, Lancashire L39 4QP UK

**Keywords:** *Aedes aegypti*, *Aedes albopictus*, AIMs, Mosquito, Surveillance, Eggs, Tyres, Dengue, Zika

## Abstract

**Background:**

The international movement of used tyres is a major factor responsible for global introductions of *Aedes* invasive mosquitoes (AIMs) (Diptera: Culicidae) that are major disease vectors (e.g. dengue, Zika, chikungunya and yellow fever). Surveillance methods are restricted by expense, availability and efficiency to detect all life stages. Currently, no tested method exists to screen imported used tyres for eggs in diapause, the life stage most at risk from accidental introduction. Here we test the efficiency of adhesive tape as an affordable and readily available material to screen tyres for eggs, testing its effect on hatch rate, larval development, DNA amplification and structural damage on the egg surface.

**Results:**

We demonstrated that the properties of adhesive tape can influence pick up of dormant eggs attached to dry surfaces. Tapes with high levels of adhesion, such as duct tape, removed eggs with high levels of efficiency (97% ± 3.14). Egg numbers collected from cleaned used tyres were found to explain larval hatch rate success well, particularly in subsequent larval to adult emergence experiments. The strength of this relationship decreased when we tested dirty tyres. Damage to the exochorion was observed following scanning electron microscopy (SEM), possibly resulting in the high variance in the observed model. We found that five days was the optimal time for eggs to remain on all tested tapes for maximum return on hatch rate success. Tape type did not inhibit amplification of DNA of eggs from three, five or ten days of exposure. Using this DNA, genotyping of AIMs was possible using species-specific markers.

**Conclusions:**

We demonstrated for the first time that adhesive tapes are effective at removing AIM eggs from tyres. We propose that this method could be a standardised tool for surveillance to provide public health authorities and researchers with an additional method to screen tyre cargo. We provide a screening protocol for this purpose. This method has a global applicability and in turn can lead to increased predictability of introductions and improve screening methods at high risk entry points.

## Background

The spread of RNA based flaviviruses and alphaviruses such as dengue (DENV), yellow fever, Zika (ZIKV) and chikungunya viruses (CHIKV) have become a major global concern. Annual infection rates of DENV (family *Flaviviridae*) have been predicted at 284–528 million [1], resulting in ~ 20,000 reported deaths per year [2]. It is a multi-form disease with varying side effects and severity, symptoms range from flu-like fevers and a characteristic skin rash, to severe haemorrhagic bleeding and potentially fatal hypertension. Some 30–54.7% (2.05–3.74 billion) of the world’s population is now believed to be at risk from infection across 128 countries [3] with little substantial progress being made in reducing the spread of both the vectors and the disease. ZIKV (family *Flaviviridae*) has also become well reported with 440,000–1,300,000 reported cases from the 2015 Brazil epidemic alone [4] and can be asymptomatic or present as mild flu-like symptoms concurrent with some DENV manifestations. ZIKV can also spread *via* intrauterine transmission leading to congenital microcephaly in unborn children [4–6], as well as Guillain-Barré syndrome [7]. Phylogenetic analysis of whole ZIKV genomes suggests the disease originated in East Africa in the 1920’s [8] and until recent pandemics across the Pacific and the Americas, had a fairly slow epidemiology [9]. Reasons for accelerated ZIKV are yet to be confirmed but are likely to be multi-causal with lack of localised immunity, increased mobility of competent vectors, delayed detection and expanding levels of globalisation the likely candidates [10, 11].

The primary vectors of these arboviruses are *Aedes* (*Stegomyia*) *aegypti* (L.) (yellow fever mosquito) and *Aedes* (*Stegomyia*) *albopictus* (Skuse) (Asian tiger mosquito). Both species have immature aquatic stages that require natural water-filled tree holes, bamboo nodes and leaf axels for egg and larval development but have also become successfully adapted to living in proximity to humans by utilising human made water-filled containers and subterranean drainage systems as viable alternatives [12–14].

The global spread of these species is associated with the transportation of human goods such as the international trade in tyres and “wet footed” plants, such as lucky bamboo [15–18]. *Aedes aegypti* and *Ae. albopictus* have biological characteristics that, to differing extents, favour invasiveness. Both have developed anthropophilic adaptive tendencies, resulting in their proximity to humans for all aspects of their life-cycle and feed primarily on human blood [19, 20]. One such adaptation includes the use of manmade containers. *Aedes aegypti* deposits 85–125 eggs [21] on the margins of small temporary pools that form within these containers where they utilise a process of diapause, an adaptation to allow maximum return for larval development in small water bodies that can quickly evaporate [22–24]. Oviposition technique is an integral part of this trait, with eggs being placed directly adjacent to the meniscus where a secretion on the exochorion adheres the egg ventrally to the material margin of the pool [25, 26]. This prevents eggs from falling into the water body and allows repeated submergence over several flooding cycles resulting in staggered larval emergence, also referred to as instalment hatching. After egg laying, a drying period of 11–13 h is required for the development of the serosal cuticle (SC), an inner membrane of the egg-shell that allows the embryo to survive desiccation [23]. In captive bred populations of *Ae. aegypti*, eggs have been reported as surviving such periods for 6–12 months depending on environmental conditions (J. Longbottom, pers. comm.). These adaptations allow aedine eggs to survive long journeys attached to the surface of vessels whilst remaining in a state of diapause. This key physiological feature has allowed for the colonisation of new territories from their ancestral origin of sub-Saharan Africa [27].

Global modelling of aedine species distribution suggest that the range of both *Ae. aegypti* and *Ae. albopictus* is still expanding, this has been particularly well recorded across European and American continents [28]. In Europe, the movement of *Ae. albopictus* has led to the first documented cases of autochthonous transmission of CHIKV and DENV [29] and reports of *Ae. aegypti* well beyond its expected range [30]. Aligned with recently recorded adaptive behaviour of AIMs [14, 31], this presents an argument for greater surveillance at the current limits of their geographical distribution [15].

The movement of AIMs appears to be multi-causal, but primarily through human transport networks. Active dispersal of these species is considered to be limited with a reported life-time mean mobility of approximately 50–363 m [32–34]. Recent evidence suggests that adults can also be passively dispersed by cars [35], the detection of *Ae. albopictus* in motorway service stations in Kent, England [36] and Bavaria and Baden-Wuerttemberg, Germany [37], supports this theory. However, the global movement of car tyres has been highlighted as a primary method of distributing *Ae. albopictus* and *Ae. aegypti* [38, 39]. It was by this method that *Ae. aegypti* was introduced into the Netherlands from Florida, USA, in 2010 [15].

To combat the further spread of AIMs international efforts have been made to increase surveillance at major ports and airports, tyre yards and service stations, as well as areas of suitable habitation [40–46].

Traditional surveillance techniques for AIMs mostly utilise oviposition-based traps, larval dipping and attractants such as CO_2_, pheromones, light and human bait [41]. The efficiency of such traps is well documented [47]. However, the deployment of different sampling methods between surveillance programmes is highly variable at an international and national scale, possibly a reflection of resource availability, as well as the varying inclination of local and national authorities to promote active surveillance [48]. These techniques have been fruitful in locating AIMs, however, these methods target larvae, pupae, adults and *in situ* egg deposition. Dormant eggs attached to the surface of car tyres, or dry containers are overlooked [15, 49]. In surveillance systems where port authority screening is relied upon as the first line of defence against AIMs, any cargo containing eggs passes freely without discovery. Additionally, current surveillance requires the presence of active females in various reproductive states. Oviposition trapping techniques rely on recently introduced females to blood feed, or to have been introduced in a gravid state. Likewise, CO_2_ and pheromone attractants require mosquitoes to be actively seeking a blood source. Such surveillance methods target adult mosquitoes that are attempting, or even succeeded, in reproducing. These methods can reduce response times for post-discovery control and often bypass populations of *Aedes* eggs being transported in a desiccated state that later emerge, when wetted, into a new territory. In this instance, early detection may only occur in countries with robust surveillance, or by chance reports of nuisance biting.

Although there is no singularly effective method of surveillance for AIMs it is essential that a range of sampling tools are available to determine their presence, to quantify the extent of any infestation and to provide accurate species identification and rapid initiation of control measures.

To develop a low cost and easy to use tool for mosquito workers to standardise screening of car tyres for *Aedes* eggs, we tested the efficacy of four distinct types of sticky tape for their ability to remove the mosquito eggs from the tyre surface. Additionally, we investigated the post-removal impact of this technique on the ability to (i) rear the sampled eggs to larvae and adulthood for identification and whether time exposed to the tape had any negative effects; (ii) identify the eggs by morphology and for possible damage, using SEM; and to (iii) investigate whether the tape types had any notable effect on DNA integrity by genotyping extracted eggs using species specific markers.

## Methods

### Maintenance of colonies

*Aedes aegypti* (New Orleans strain) laboratory colonies were reared within the Edge Hill University Vector Biology Research Group insectaries at 27 °C and 70% RH, on an 11 h day/night cycle with a simulated 60 min dawn/dusk period, using a lighting system of 4× Osram Dulux 26W 840 lights positioned approximately 2 m from the rearing cages. Several hundred eggs (~ 1000–1500) deposited on filter paper were stimulated to hatch by submerging filter papers in a broth of 0.1 g brewer’s yeast (Holland & Barrett, Ormskirk, UK) and 0.5 g of nutrient broth (Sigma-Aldrich, Dorset, UK) dissolved in 1.4 l of dH_2_O [50]. Of those successfully hatched (~ 500–1000), first- and second-instar larvae were separated into 4 or 5 separate trays to avoid overcrowding and fed using ground fish flakes (Aquarian Tropical Fish Food, UK), ~ 0.08 mg per larvae after hatching with volumes doubled for each day there after until pupation. All fourth-instar larvae and pupae were then transferred into 30 × 30 × 30 cm insect rearing cages (#211261, BugDorm-1. NHBS, Totnes, UK) and emergent adults fed for the first three days on 10% sugar solution soaked into cotton wool. Emergent male and female mosquitoes were allowed to mix for a minimum of three days to ensure that most females had the opportunity to copulate. After three days, all females (~ 400–500) were removed and placed into separate rearing cages and starved for 24 h prior to blood-feeding. All females were engorged on defibrinated horse blood (Thermo Fisher Scientific, Altrincham, UK) using the Hemotek^®^ membrane feeding system (Hemotek Ltd, Blackburn, UK) until fully distended and then left for 24 h with a supplement of 10% sugar solution until gravid. Any gravid females were then separated into two equal batches; the first batch was used within experimental treatments (~ 200) and the second batch for rearing the next generation (~ 200). Specimens used during experimentation did not exceed more than three generations to reduce possible effects of inbreeding depression on egg viability [51].

### Egg laying on car tyres

**Fig. 1:**
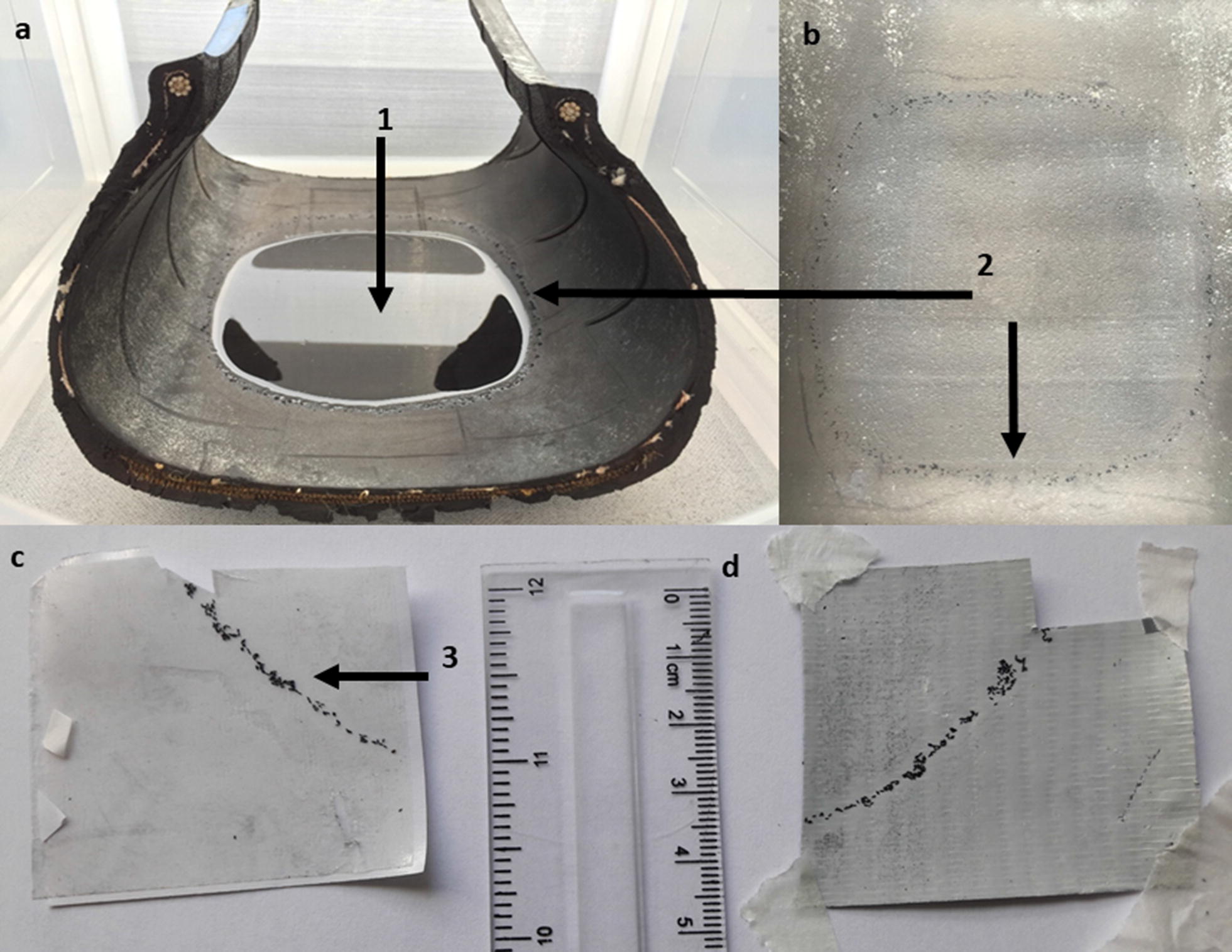
Visibility of Ae. aegypti egg deposition on tyre and tape sections. **a** Section of tyre placed within a bugdorm. **b** Dorsal view of the tyre after drying. **c** After sampling, eggs attached to adhesive surface of double-sided carpet tape. **d** After sampling, eggs attached to adhesive surface of duct tape. (1) 75 ml of dH_2_0 added to the tyre to encourage oviposition. (2) The deposited eggs can be seen by the naked eye as a dark speckled ring around the margin of the reservoir. (3) Increased visibility of eggs on adhesive tape after sampling

Two used car tyres (175/70 R13, Michelin, UK) were obtained from an outdoor store at a farmyard in Burscough, Lancashire and each divided into eight equal sections. A first batch of eight sections were scrubbed with a detergent, sterilised with bleach and thoroughly rinsed using dH_2_O to ensure no residual contaminants were present (hereafter referred to as ‘clean’). A further eight were left in the condition they were found in (hereafter referred to as ‘dirty’). Each section was ~ 21 (L) × 17 (W) × 13 (H) cm in size, just large enough to move in and out of bugdorms without dislodging or disturbing *in situ* eggs. Four tyre sections were used per round of egg laying with a total of 16 replicates/tape type/treatment (treatment = no. of days exposed to the tape). For each replicate, a section of car tyre was added to each rearing cage along with 20 gravid females and 75 ml of dH_2_O deposited into the centre of each tyre section to create a small pool in the central depression. The aim here was to encourage female oviposition and egg adherence around the margin (Fig. 1). Females were left for 72 h to lay eggs and then removed by aspiration. The remaining dH_2_O was drawn from each tyre by pipetting to prevent dislodging the eggs and ensure that they were not stimulated to hatch. Tyre sections were then removed from the bugdorm and allowed to dry at room temperature for 24 h.

### Egg sampling/larval rearing with sticky tape

Four tape types were selected based on different properties (summarised in Table 1). Each tape type was cut into a 50 mm^2^ sections and applied to the tyre along the line of deposited eggs, using a stratified random sampling method and adhered with gentle pressure from the tip of the index finger. Tape application was carried out by the same two individuals to reduce possible user bias. The outline of each piece of tape was marked using white chalk. Each tyre section accommodated 8 × 50 mm^2^ pieces of tape allowing for two replicates of each tape type per tyre section. The tape was removed using fine tipped entomological forceps and placed into a dry 250 ml glass beaker and counted under stereomicroscope (Leica M80, GT vision, Suffolk, UK) at 40× magnification, along with any remaining eggs within the marked areas on the tyre.

**Table 1:** Properties of adhesive tapes used during experimentation

Tape type	Code	Properties	Cost
Body tape	BT	Medium levels of adhesion. Less commercially available but contains less chemicals that potentially have less deleterious effects on egg viability	£4.90 for 27 strips (Eylure Body Tape Pre-Cut Adhesive Strips, Boots UK, Ormskirk, England)
Clear packing tape	PT	Low-medium levels of adhesion, wide commercial availability, low cost, may be less damaging to eggs during transfer	£3.28 for (L) 100 M (W) 50 mm (Diall Clear Packing Tape, B&Q, Aintree, England)
Double-sided flooring tape	CT	Medium-high levels of adhesion. Widely available in DIY stores, double-sidedness may prove advantageous for adhering samples for transportation	£8.90 for (L) 25 M (W)50 mm (Diall White Double Sided Tape, B&Q, Aintree, England)
Duct tape	DT	High levels of adhesion, wide commercial availability, a white background to improve visibility, easily torn without the need for cutting apparatus	£ 2.70 for (L) 5 M (W) 50 mm (Duck Tape®, B&Q, Aintree, England)

Three egg exposure treatments of three, five- and ten-day intervals were undertaken to determine if the tapes had any effect on hatch rate success. After the allotted exposure time, the egg-laden tapes were submerged in 100 ml of hatching broth in a 250 ml beaker. Emergent larvae were counted after 24 h and transferred into beakers with 100 ml of dH_2_0 and reared as described above. A laboratory control was also established whereby the same number of gravid females were encouraged lay on filter paper and put through the same treatments as applied to the different tape types.

After the first 24 h of submergence, tapes were removed and dried at room temperature for 48 h and re-submerged for a second time. Larvae were once again removed and counted after 24 h. All larvae were left to develop into adults and numbers recorded to determine if tape exposure effected later development.

### Scanning electron microscopy (SEM)

A descriptive approach was used to observe potential damage to the egg morphology caused by the removal of eggs using tape by making observations through scanning electron microscopy (6010LV, JEOL (UK) Ltd., Herts, UK). Sections (10 mm^2^) of egg loaded tape of each type and treatment, plus controls, was cut away and attached to 12.5 mm SEM stubs using carbon tabs (Agar Scientific Ltd., Essex, UK). Samples were then coated in gold for 4 min (~ 4 nm) using a sputter coater (Q150R ES, Quorum Technologies Ltd., East Sussex, UK). Eggs were left *in situ* throughout this process. Samples were viewed in high vacuum mode where accelerated voltage = 10 kV, WD = 12 and spot size = 50.

### Molecular methods: PCR-based identification

Additional checks were made do determine if amplification of egg DNA was still possible after three, five and ten days attached to the different tape types. A subsample (10 mm^2^ squares) from each tape type, exposure and treatment were removed from egg-loaded tapes and placed into individual 1.5 ml tubes and stored at − 20 °C until extraction. The number of eggs in each subsample ranged from 2 to 16. Each sample was homogenised (including the tape) for 30 s whilst dry using an electronic pestle and mortar (431–0094, VWR, Leicestershire, UK), 180 µl of buffer ATL and 20 µl of proteinase K (Qiagen, Manchester, UK) was added and homogenised for several seconds. Samples were thoroughly vortexed and left to lyses on an orbital shaker at 56 °C for 15 h after which when the remaining pieces of tape were removed from each sample and discarded. DNA extraction was completed using the DNeasey^®^ Blood and Tissue Spin Column Kit (Part no. 69506, Qiagen, Manchester, UK) following the provided protocol, with 50 µl elution buffer held in the columns for 5 min and passed through the column twice to increase DNA yield. To test amplification, a polymerase chain reaction (PCR)-based analyses was carried out using species-specific primers developed by Das et al. [52] and Higa et al. [53] (Table 2).

**Table 2:** Species-specific PCR primers for the identification of Ae. aegypti

Species	Primer code	Sequence (5ʹ-3ʹ)	Reference
Universal forward primer	AUF	TCAAAATTAAGGGTAGTGGT	[52]
Universal reverse primer	AUR	GACTTCAACTGGCTTGAACT	[52]
Ae. aegypti	AEG	GACACCGAGGCGCCCATTGC	[52]
Universal forward primer	18FHIN	GTAAGCTTCCTTTGTACACACCGCCCG	[53]
Universal reverse primer	CP16	GCGGGTACCATGCTTAAATTTAGGGGGTA	[53]
Ae. aegypti	aeg.r1	TAACGGACACCGTTCTAGGCCCT	[53]

Each 25 µl PCR reaction for the Das et al. [52] primers consisted of 2.5–20 ng of DNA template, 0.5 U of Phusion® High-Fidelity Polymerase (New England Biolabs® Ltd. Herts, UK), 1× Phusion HF Buffer (NEB), 200 µm of dNTP mix (NEB), 0.5 µM of primers AUF and AUR and 0.7 µM of AEG and 2% DMSO (NEB). PCR amplification was performed using a Primer Thermal Cycler (Techne, Staffordshire, UK) programmed with an initial denaturation of 98 °C for 30 s, followed by 35 cycles of 98 °C for 10 s (denaturation), 59 °C for 20 s (annealing), 72 °C for 20 s (extension), followed by a final extension of 72 °C for 7 min. Each 25 µl PCR reaction for the Higa et al. [53] primers consisted of 2.5–20 ng of template, 0.5 U of Phusion® HF Polymerase, 1× Phusion HF Buffer, 200 µm of dNTP mix, 0.5 µM of primers 18SFHIN and CP16 and 0.7 µM of aeg.r1. Thermal cycler setting as outlined above, but with an annealing temperature of 70 °C. PCR products were resolved on a 2% agarose/ethidium bromide gel with HyperLadder™ 100 bp (Bioline Reagents Ltd, London).

### Analysis

A comparison of the egg pick-up efficiency of the different tape types was carried out using a Kruskal–Wallis test, after a Shapiro–Wilks test showed that the data were non-parametric. Multiple comparisons between tape types for the egg pick-up efficiency was investigated using the Nemenyi *post-hoc* test.

To test how the number of larvae and adults was affected by the number of eggs and larvae, respectively and in regard to different tape types and egg exposure time to the tape (i.e. 3, 5 and 10 days), mixed effect models were used. We selected the best-fit model using Akaikeʼs information criterion (AIC) and from there we calculated total explained variance for each fixed and random effect term. In addition, we used linear models to investigate individual tape treatments and to test the correlations between the number of eggs picked up and larval hatch rate and number of larvae and emergent adults. A Shapiro–Wilks test showed that the hatch rate and adult emergence data were non-parametric, henceforth we square root transformed the data before running the linear models. To account for differences in the starting number of eggs and larvae for each sample, we weighted the transformed data proportionately by the total number of adults in the population and accounted for differences of larval success from the previous treatments as follows:

$$weight = \left( {\left( {\frac{{A_{1} }}{{\sum A_{Total} }}} \right)A_{1 + 2} - L_{1 + 2} } \right) - 1$$

where A_1_ is the number of adults in a sample following the first emergence, A_Total_ is the total number of adults in the population, A_1+2_ is the number of adults in a sample following first and second emergence and L_1+2_ is the number of larvae in a sample following first and second hatching. Analyses was carried out using RStudio v.3.4.1.

## Results

**Fig. 2:**
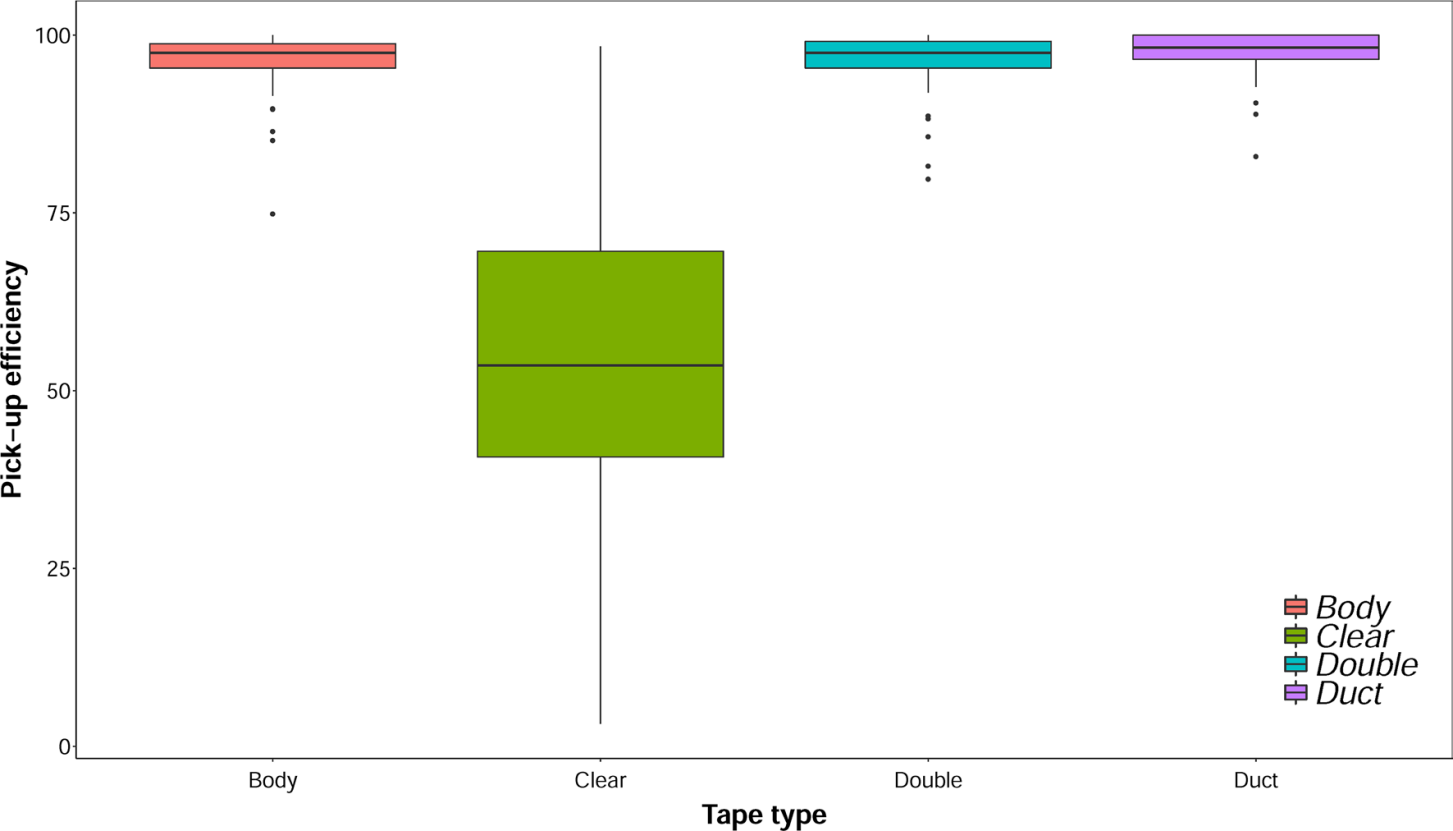
Box plots with error bars of tape types vs percentage egg pick-up from the surface of clean tyres

Within the parameters of the experiment 41,337 eggs were laid in total and across all tyre replicates (eggs, *n* = 29,170) and filter paper controls (eggs, *n* = 12,167). A total of 25,670 (88%) eggs were picked up by all tape type replicates, 11,056 (43.07%) of tape treatments and 7771 (63.87%) of controls developed into first-instar larvae during first submergence. The second submergence produced 1469 (5.04%) larvae from tape treatments and 61 (0.55%) from controls, 10,808 (51.06%) first submergence eggs successfully hatched from clean tyres replicates, 1033 (4.88%) from second submergence. A considerably lower hatch-rate was recorded for samples collected from dirty tyres; 248 (5.51%) of first submergence eggs were hatched and 436 (9.68%) from the second submergence (Table 3). Analysis of the egg pick-up efficiency using a Kruskal–Wallis test showed significant differences between the efficiency of tape types (*χ*^2^ = 114.52, *df* = 3, *P* < 0.0001). Further exploration of the data using a *post-hoc* Nemenyi test demonstrated that the difference in pick-up efficiency was between the PT and all other tapes. There were no statistically significant differences between all other treatments (Table 4). Notably, the egg pick-up efficiency of DT, BT and CT showed high levels of egg pick up efficiency (> 96%) and relatively low levels of variation (s = 3.14–1.39) compared to that of PT (55.18%, s = 22.19) (Fig. 2).

**Table 3:** Synopsis of data testing tape pick-up efficiency, larval hatch rate and adult emergence

Tape type	Exposure^a^	Treatment	Results									
			Egg pick-up		Larval hatch rate (1st sub.)		Larval hatch rate (2nd sub.)		Adult emergence (1st sub.)		Adult emergence (2nd sub.)	
			n	Mean ± SD_(n-1)_ (%)	n	Mean ± SD_(n-1)_ (%)	n	Mean ± SD_(n-1)_ (%)	n	Mean ± SD_(n-1)_ (%)	n	Mean ± SD_(n-1)_ (%)
BT	3	Clean	1517	95.22 ± 4.23	428	27.65 ± 22.11	134	15.87 ± 15.18	341	83.54 ± 17.14	97	69.89 ± 25.83
PT	3	Clean	651	38.78 ± 19.43	83	13.92 ± 14.80	60	6.08 ± 7.13	68	94.25 ± 24.18	34	74.55 ± 23.74
CT	3	Clean	1943	92.13 ± 5.86	414	23.84 ± 21.10	281	22.27 ± 16.81	342	81.75 ± 12.65	205	81.75 ± 16.56
DT	3	Clean	1897	95.36 ± 4.52	397	20.94 ± 25.10	304	24.19 ± 15.02	292	70.45 ± 32.44	117	44.02 ± 28.10
na	3	Control	na	na	2713	64.14 ± 8.71	24	3.15 ± 3.79	2131	81.52 ± 15.31	21	36.11 ± 45.26
BT	5	Clean	1807	94.97 ± 6.22	1294	67.74 ±19.71	66	6.32 ± 19.91	1112	84.95 ± 10.97	62	89.66 ± 22.18
PT	5	Clean	1130	56.42 ± 13.75	800	69.45 ± 7.99	17	4.08 ± 13.02	692	85.14 ± 8.17	17	100
CT	5	Clean	2098	97.43 ± 1.72	1392	64.56 ± 26.52	158	8.67 ± 21.63	1209	87.94 ± 6.32	152	98.84 ± 2.10
DT	5	Clean	2324	97.78 ± 2.39	1818	76.75 ± 6.66	7	2.13 ± 4.60	1453	81.25 ± 11.52	7	100
na	5	Control	na	na	2592	69.35 ± 9.77	24	4.49 ± 5.38	2119	83.22 ± 11.24	15	55 ± 44.44
BT	10	Clean	2059	97.47 ± 2.39	1082	49.24 ± 23.15	1	0.12 ± 0.48	964	87.9 ± 10.16	0	0
PT	10	Clean	1220	52.64 ± 15.78	577	50.58 ± 26.56	2	0.08 ± 0.33	535	89.7 ± 11.82	0	0
CT	10	Clean	2135	98.01 ± 1.53	1206	55.33 ± 25.09	0	0	1038	86.2 ± 4.27	na	na
DT	10	Clean	2387	98.18 ± 1.22	1317	54.52 ± 26.28	3	0.18 ± 0.74	1163	86.12 ± 11.35	3	100
na	10	Control	na	na	2466	59.42 ± 7.93	13	4.75 ± 5.85	2089	86.5 ± 7.22	13	100
BT	5	Dirty	1918	98.77 ± 1.06	82	4.35 ± 4.51	153	6.94 ± 4.76	30	72.08 ± 47.70	64	43.25 ± 22.63
PT	5	Dirty	499	90.57 ± 7.62	24	3.79 ± 3.04	71	12.15 ± 9.12	23	85.71 ± 37.80	50	69.94 ± 26.17
CT	5	Dirty	1212	98.97 ± 2.37	73	6.44 ± 4.81	109	8.95 ± 6.06	66	92.6 ± 7.83	55	45.96 ± 19.90
DT	5	Dirty	873	99.49 ± 1.44	69	7.92 ± 8.14	103	11.82 ± 6.37	24	66.23 ± 43.11	31	37.38 ± 36.57

Notes: Data testing tape pick-up efficiency, larval hatch rate based on first and second submergence of tapes from egg pick-up and adult emergence from successfully reared larvae (including controls)

^a^Exposure: no. of days that eggs were exposed to the tape

Abbreviations: n, total egg pick-up across all samples; SD, standard deviation from the percentage mean; na (within egg pick-up) where eggs were hatched directly from filter paper controls; na (within adult emergence) where no adults could emerge due to zero hatch-rate scores

**Table 4:** Egg pick-up efficiency in different sticky tape treatments. Results of the Nemenyi post-hoc test following a Kruskal–Wallis test

Tape type	Egg pick-up (%)	Tape type			
	Mean ± SD	BT	PT	FT	DT
BT	96.30 ± 4.39	–	–	–	–
PT	55.18 ± 22.19	5.2e−14	–	–	–
CT	96.3 ± 4.34	1.00	4.9e−14	–	–
DT	97.45 ± 3.14	0.54	3.6e−14	0.59	–

Abbreviation: SD, standard deviation

### The effect of tape type on hatch-rate success and adult emergence

**Fig. 3:**
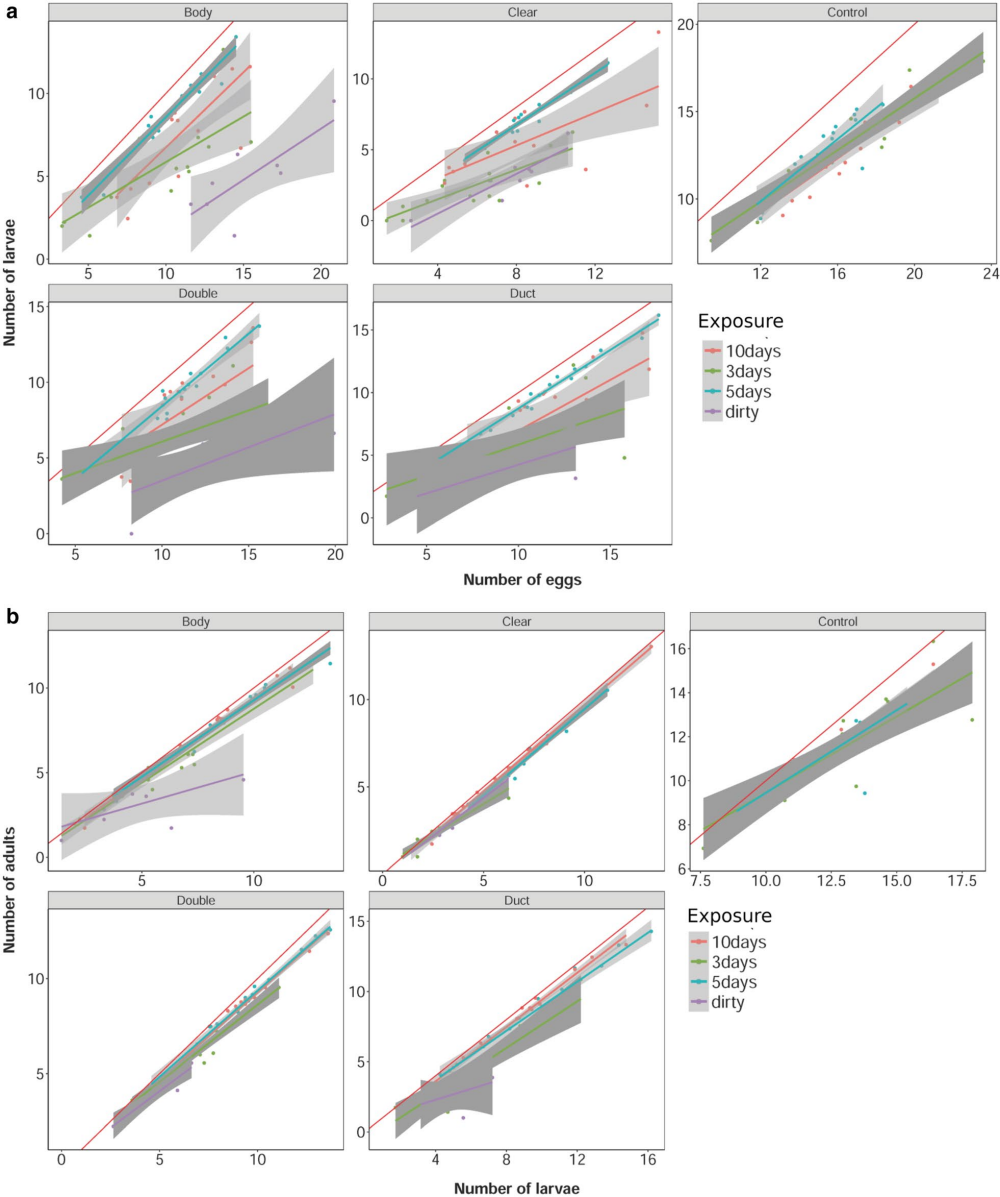
Visualisation of linear model R^2^ data from hatch rate success of eggs picked up by tape types and subsequent adult emergence. **a** Visualised linear model of the hatch rate success of eggs picked up by different adhesive tape treatments. **b** Adult emergence from those successfully hatched

To test if the number of eggs picked up can be used to explain the number of larvae hatching and whether the number of larvae hatching can explain the number of emergent adults between the different tape types and days of exposure, two approaches were used. In the case of hatch rate success (i.e. the transition from eggs picked up to hatched larvae) the best-fit mixed effect model (*R*^2^ = 0.77) included number of larvae and time of exposure as a fixed and tape type as a random factor (Tables 5, 6). Similarly, in the case of adult emergence (i.e. the transition of hatched larvae to adult) the best-fit mixed effect model (*R*^2^ = 0.99) included number of larvae and time of exposure as a fixed and tape type as a random factor (Tables 5, 6). In both models, most of the variance was explained by the number of eggs (*R*^2^ = 0.64) or the number of larvae (*R*^2^ = 0.97, Table 6). This was followed by tape type and exposure (Table 6). To investigate the effect of individual tape types a linear model was fitted for each treatment combination (Fig. 3, Tables 7, 8). The results indicated that the number of larvae that hatch is a good indicator of the number of adults that will emerge (Table 5). All treatments fell close to the 1:1 line, except for BT and DT from the dirty tyres. Therefore, if eggs hatch, it is likely that they will make it to adulthood; however, the number of eggs less efficiently explained the number of successfully hatched larvae. Generally, the five-day treatment resulted in stronger correlations compared to the three- and ten-day treatments (Tables 7, 8, Fig. 3). Regardless of the length of exposure to the tapes, the dirty tape always resulted in poorer explanatory power of either emerged larvae number from egg number, or adult emergence number from larvae number.

**Table 5:** Mixed effect model results of number of eggs hatching and the numbers of larvae to adult emergence for the different tape types and exposure treatments

Development transition	Model	Form	df	AIC	BIC	P-value
Egg to larvae emergence	M1	No. of larvae ~ No. of eggs + Exposure + (Tape type)	7	2635.1	2660.3	2.00E−16***
	M2	No. of larvae ~ No. of eggs + Exposure	6	2647.4	2669.0	2.00E−16***
	M3	No. of larvae ~ No. of eggs + (Tape type)	4	2757.9	2772.3	1
	M4	No. of larvae ~ Exposure + (Tape type)	6	2882.8	2904.4	1
	M5	No. of larvae ~ Tape type	6	2908.8	2930.5	1
	M6	No. of larvae ~ Exposure	5	3016.0	3034.1	1
Larvae to adult emergence	M7	No. of adults ~ No. of larvae + Exposure + (Tape type)	7	684.3	709.5	2.00E−16***
	M8	No. of adults ~ No. of larvae + Exposure	6	747.4	769.1	2.00E−16***
	M9	No. of adults ~ No. of larvae + (Tape type)	4	706.4	720.9	1
	M10	No. of adults ~ Exposure + (Tape type)	6	2861.1	2882.7	1
	M11	No. of adults ~ Tape type	5	2998.1	3016.1	1
	M12	No. of adults ~ Exposure	5	2998.1	3016.1	1

Note: The model with the lowest AIC (Akaikeʼs information criterion) and BIC (Bayesian information criterion) was retained (i.e. the best-fit model) and Chi-square test was used to test for significance

*P < 0.05, **P < 0.01, ***P < 0.001

**Table 6:** Summary of the variance from the models that best explained eggs hatching and the numbers of larvae to adult emergence

Development transition	Variable	Effect	Explained variance (Adj. R^2^)	Unexplained variance (Adj. R^2^)
Egg to larvae emergence	Total variance		0.77	0.23
	No. of eggs	Fixed	0.64	0.36
	Tape type	Random	0.42	0.58
	Exposure	Fixed	0.14	0.86
Larvae to adult emergence	Total variance		0.99	0.01
	No. of larvae	Fixed	0.97	0.03
	Tape type	Random	0.43	0.57
	Exposure	Fixed	0.14	0.86

Note: For each contributing variable (i.e. tape type and exposure) the explained and the unexplained are presented

**Table 7:** Linear model results of tape type and exposure vs hatch rate success of eggs from egg pick-up

Tape type	Exposure (days)	R^2^	F	df	Residual SE	P-value
Control	3	0.8926	125.6	14	1.233	2.24E−08***
BT	3	0.5537	16.6	14	2.514	5.73E−04***
PT	3	0.6197	25.4	14	1.683	1.79E−04***
CT	3	0.4160	11.7	14	2.604	4.16E−03**
DT	3	0.3552	9.3	14	3.021	8.76E−03***
Control	5	0.7588	48.2	14	1.015	6.84E−06***
BT	5	0.9509	291.2	14	0.649	9.14E−11***
PT	5	0.9565	331.1	14	0.408	3.86E−11***
CT	5	0.9156	163.7	14	0.647	4.09E−09***
DT	5	0.9793	711.8	14	0.463	2.10E−13***
Control	10	0.8934	126.7	14	1.001	2.11E−08***
BT	10	0.5921	22.8	14	1.662	2.98E−04***
PT	10	0.4102	11.4	14	2.508	4.49E−03**
CT	10	0.3118	7.8	14	2.062	1.44E−02*
DT	10	0.3967	10.9	14	2.047	5.30E−03***
BT (Dirty)	5	0.5239	8.7	6	2.112	2.56E−02*
PT (Dirty)	5	0.7902	27.4	6	1.063	1.95E−03**
CT (Dirty)	5	0.4160	6.0	6	2.728	5.00E−02
DT (Dirty)	5	0.3870	5.4	6	2.227	5.88E−02

*P < 0.05, **P < 0.01, ***P < 0.001

Abbreviations: df, degrees of freedom; SE, standard error

**Table 8:** Linear model results of tape type and exposure vs adult emergence success from hatched larvae

Tape type	Exposure (days)	R^2^	F	df	Residual SE	P-value
Control	3	0.7052	36.9	14	1.528	2.87E−05***
BT	3	0.9493	282.0	14	0.596	1.13E−10***
PT	3	0.9436	252.1	14	0.417	2.39E−10***
CT	3	0.9549	318.6	14	0.429	4.99E−11***
DT	3	0.7707	51.4	14	1.389	4.78E−06***
Control	5	0.7629	49.3	14	0.992	6.04E−06***
BT	5	0.9765	624.9	14	0.427	5.13E−13***
PT	5	0.9764	622.1	14	0.270	5.29E−13***
CT	5	0.9734	550.1	14	0.355	1.23E−12***
DT	5	0.9436	251.8	14	0.699	2.41E−10***
Control	10	0.9564	330.3	14	0.502	3.92E−11***
BT	10	0.9802	744.4	14	0.413	1.54E−13***
PT	10	0.9939	2432.0	14	0.209	2.00E−16***
CT	10	0.9941	2518.0	14	0.232	2.00E−16***
DT	10	0.9823	831.2	14	0.415	7.22E−14***
BT (Dirty)	5	0.2725	3.6	6	2.005	1.06E−01
PT (Dirty)	5	0.9797	338.9	6	0.254	1.66E−06***
CT (Dirty)	5	0.9671	206.6	6	0.391	7.10E−06***
DT (Dirty)	5	0.4051	5.8	6	1.433	5.32E−02

*P < 0.05, **P < 0.01, ***P < 0.001

Abbreviation: SE, standard error

### Cuticular condition and species identification by SEM

**Fig. 4:**
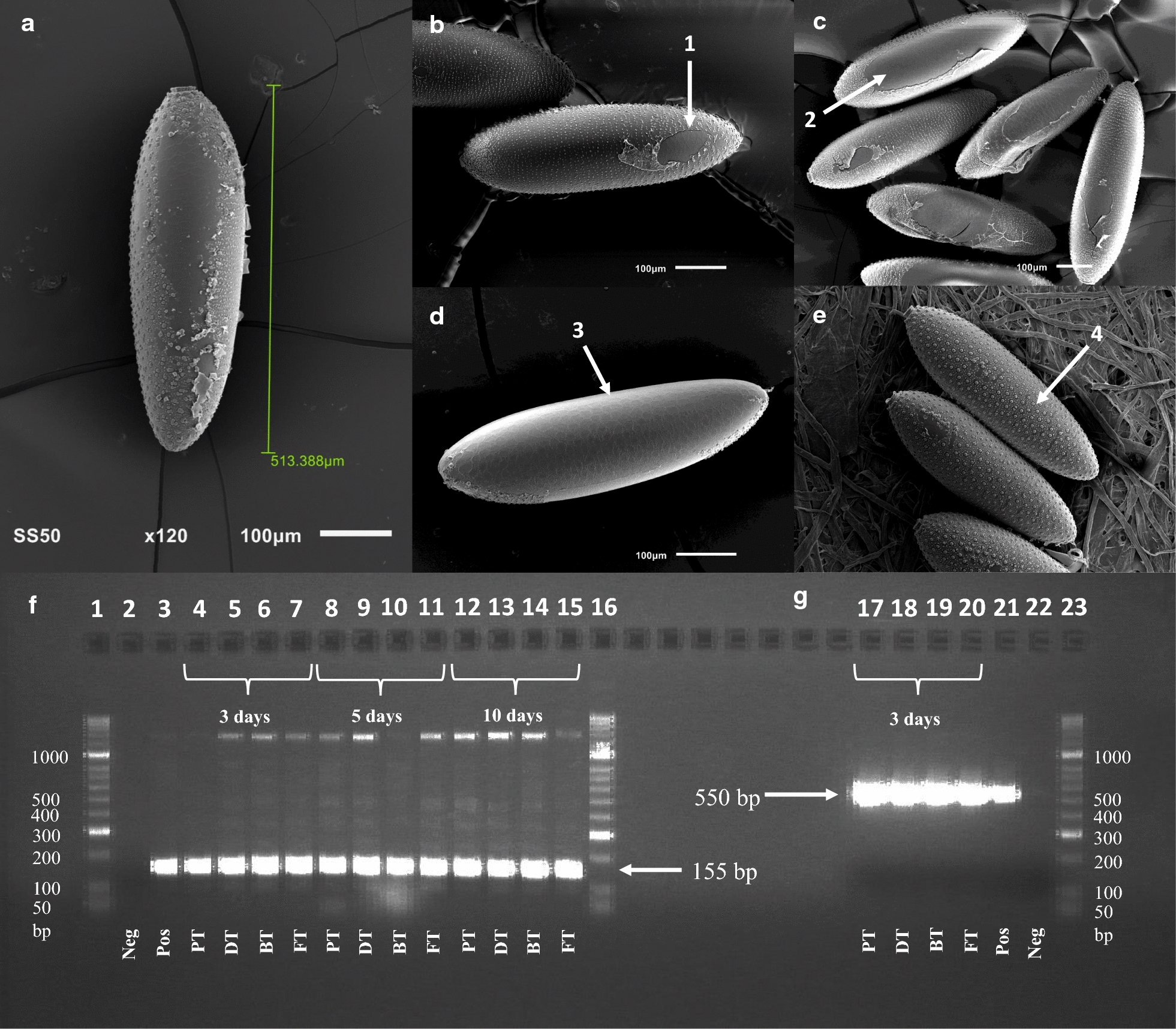
Visualisation of DNA amplification and egg damage caused by egg removal. PCR amplification of Ae. aegypti using species-specific markers and visualisation of tape damage to the egg surface under SEM. a–e SEM micrographs of Ae. aegypti mosquito eggs. a Example of egg size, dorsal view, lifted from filter paper. b Dorsal view on BT (1, damage to chorion). c Dorsal view on DT (2, damage to chorion). d Dorsal view on FT (3, total removal of chorionic layer). e Ventral view, controls on filter paper (4, chorion intact). Gel electrophoresis results of PCR of eggs from car tyres using primers of Higa et al. [50] (f) and those of Das et al. [49] (g)

Descriptive observations were made of the condition of the eggs *in situ* upon both the control and tape treatments using SEM revealing that most of the eggs removed from car tyres showed varying levels of damage to the exochorion (Fig. 4), but less so to the endochorionic and serosal cuticle [54]. Additionally, SEM micrographs demonstrate that identification of the mosquito eggs *via* morphology alone is unlikely to be possible using this sampling technique as only a dorsal perspective of the egg is visible after pick-up.

### Interference of DNA amplified from mosquito eggs

DNA isolation from mosquito eggs following the use of tape produced DNA yields suitable for successful PCR amplification. Isolation from a single egg extracted from clear tape should produce enough yield to amplify species-specific markers that could be easily visualised with ethidium bromide gel electrophoresis. All treatments including from controls successfully amplified using the species-specific markers indicating that tape treatment does not inhibit amplification of DNA after a period of up to 10 days of exposure to tape types followed by storage at − 20 °C.

## Discussion

### Identification of aedine species from eggs

Adhesive tape has previously been used in the trapping of other adult insects [55, 56] and the application of adhesive tape to locate mosquito eggs is not unknown between entomologists working in the field, although it is only rarely reported in grey literature [41]. We find no validation of tape in surveillance or ecological studies of mosquito eggs. It is probable that the development of adhesive tapes for this purpose has been overlooked, as the identification of mosquito eggs to the species level was historically carried out by morphology, often difficult and required an elevated level of expertise with often ambiguous results. However, the recent increase in accessibility, doubled with reduced costing for technologies, such as matrix assisted laser desorption/ionization (MALDI-TOF) [57], scanning electron microscopy (SEM) [58] and genetic techniques such as species-specific markers [52, 53, 59], DNA barcoding [60, 61] and eDNA analysis [62], has eased the burden of egg identification. Rearing techniques for *Aedes* has also improved over the last 50 years with many publications providing a myriad of workable methodologies [50, 63–65].

The rapid development of such new species identification systems must be matched with a progressive approach to field sampling techniques. Surveillance for eggs is advantageous as it allows for a timelier response to introductions.

### Adhesive tape in *Aedes* surveillance

Currently 50% of the world’s population is at risk from DENV due to the presence of AIMs such as *Ae. aegypti* and *Ae. albopictus*. Aligned with this, the threat of autochthonous transmission of arbovirus diseases (e.g. DENV, CHIKV and ZIKV) in areas where such diseases are not endemic is becoming a serious public health issue [29, 66–70]. The international movement of AIMs is a serious threat to human health and it is vital that the scientific community and public bodies, develop novel methods of surveillance to increase efficiency. Here, we have demonstrated the value of adhesive tape as a method of improving surveillance by assisting in the identification of tyres that carry AIM eggs in diapause. Adhesive tape has a global availability and can be acquired inexpensively, making it a readily available material for use in tyre screening at any location around the world. However, the application of this method must be carefully considered before use in the field. Locating mosquito eggs *via* the application of adhesive tape is not a cause to assume that AIMs, or those with vector potential have been located. Eggs could be in fact from container-breeding, non-vector, species of mosquitoes. Therefore, understanding the viability of eggs after taping for downstream processing, such as rearing and identification, is essential. Additionally, the use of such tapes to sample dirty tyres in the field could result in unpredictable variance in hatch rate success when using a rearing approach for species identification. Therefore, we do not discourage but suggest caution when using this approach until field validation of this method is undertaken.

### Adhesive quality effect on egg pick-up

From the data, we can infer that all tape types were able to pick up mosquito eggs from the surface of tyres with differing levels of efficiency. The tapes with greater adhesive strength (BT, DT, CT) were able to remove most of the eggs from any given area consistently, whereas clear packaging tape (PT), with the lowest adhesion of all those tested, proved to be significantly more variable suggesting that adhesive quality of tape is important if a surveillance strategy requires accurate population census information, but less important if only ascertaining presence/absence.

### Rearing from collected eggs

The data collected shows that if a larva hatches from an egg collected by adhesive tape then it is likely to make it to adulthood. The success of hatching from eggs is much more variable and has a lower explanatory power than the controls. This suggests a possible underlying effect caused by egg removal using tape and could be explained by damage to the egg cuticle observed using SEM. However, mean hatch rate success over 5 and 10 days of exposure was above 50% for the first submergence, therefore this method is still useful for surveillance despite the negative impact caused by the sampling method. If only a small number of eggs are recovered during sampling, then we would advise a cautioned use of the rearing method.

This experiment also included a test on a set of dirty tyres with 5 days of exposure. Results showed that dirty tyres resulted in a lower explanatory power of the number of larvae from the number of eggs when using different tapes. It is likely that this is the result of (i) contamination of the tested tyres by an unknown introduced pathogen at source, or (ii) damage to the egg structure resulting in infection vulnerability from the unclean surface, or a loss in the ability to retain internal humidity. Although these hypotheses are plausible explanations for our results, further work using soiled tyres would be required to assess this.

We suggest that if the only facilities available for egg identification is through the rearing of larvae to adult, a tape with lower adhesive qualities is preferable, but will likely come at a cost of the total percentage of eggs picked up in a given area. As previously mentioned, caution should be taken when sampling dirty tyres in the field, as our result suggest it could affect both pick-up and hatch rate success.

In the instance where larval rearing is the preferred approach, our model showed that using egg number to explain larvae number, or larvae number to explain adult number, is highest (highest *R*^2^) at 5 days. Therefore, larval rearing at 5 days from the date of sampling would be optimal. The experiments tested here are from a laboratory-based study only, during field application, eggs are likely to have been attached to the tyre surface for an unknown period producing an additional unpredictability factor when estimating hatch rate success in wild sampled populations. It would be interesting to test egg viability in combination with our methods based on actual shipping conditions (e.g. duration of the journey, predicted climate condition inside the containers, etc.) in order to develop a predictability model on the likelihood of emergent risk of AIMs.

### PCR-based identification

Where larval/adult rearing is not practical, or a faster method of species identification is required, species genotyping is a viable alternative. We tested this method using egg loaded tape sections from each of the treatments to observe whether amplification was possible after exposure to the tape surface. A targeted species-specific approach was chosen as a preferable method to eliminate amplification from potential sources of contamination from the surface of the tyres. We would recommend that similar tests are undertaken during any field trials that expand this sampling method. However, there have been only several targeted species-specific assays produced for regions that are inflicted with a unique collection of problematic species [52, 53, 59, 69–73]. Alternative methods of species identification using eDNA, or the application of metabarcoding could prove to be a valuable alternative to species specific assays but will require further development.

## Conclusions

**Fig. 5:**
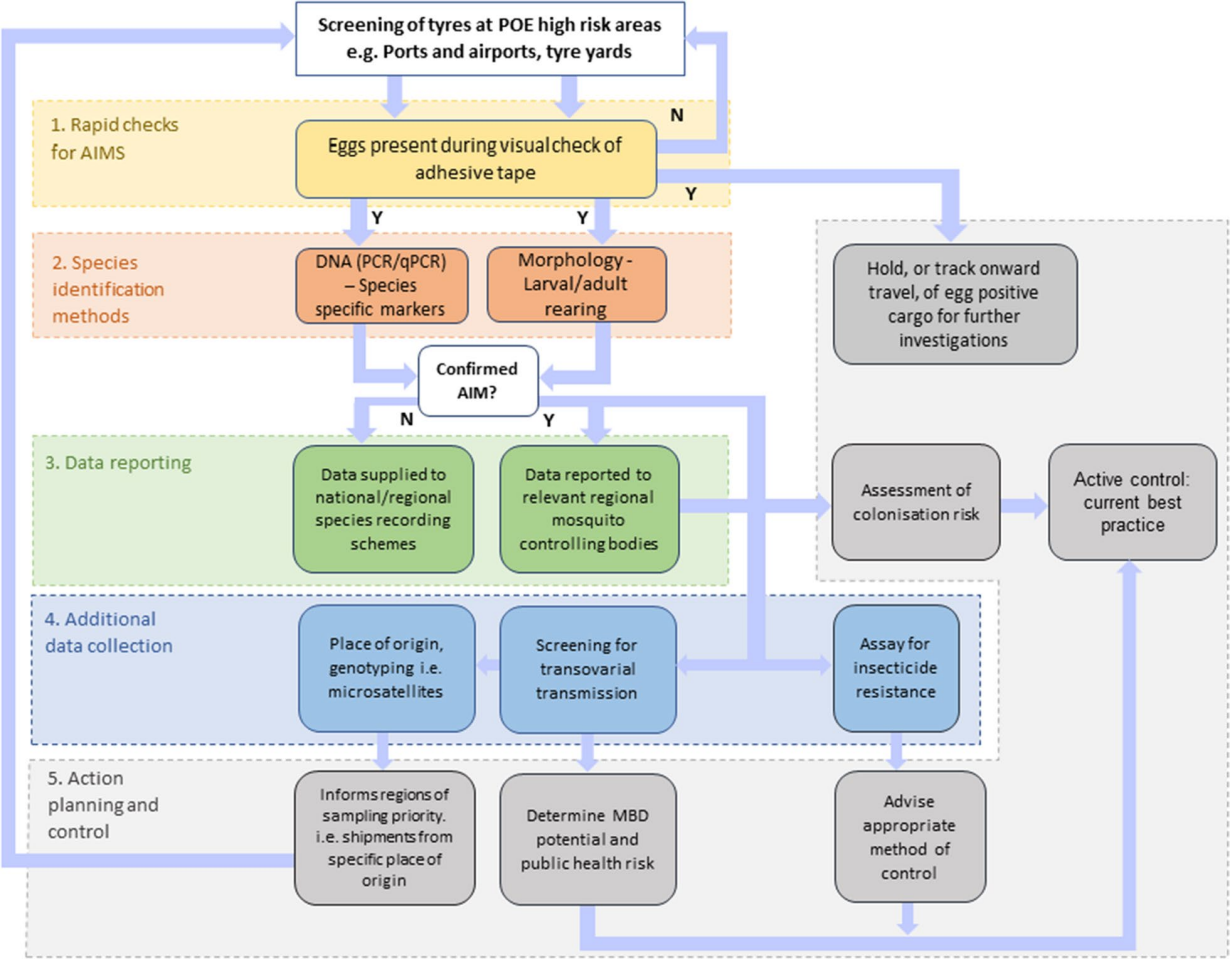
Flow diagram of a recommended procedural process for screening car tyres with adhesive tape. Abbreviations: POE, point of entry; AIM, Aedes invasive mosquito; MBD, mosquito-borne disease

This study has shown that a method of screening used tyres for mosquito eggs with sticky tape could prove to be a useful tool in the surveillance of AIMs that pose a serious threat to human health. Despite the global threat, there is currently no surveillance technique that screens for the presence of AIM eggs as a primary introduction route. Identification at this point is important as AIMs have proven to be biologically adaptive to new conditions and are successfully invasive in many areas around the world [19, 20]. This study has demonstrated that low-cost adhesive tape can be used to detect the eggs of *Ae. aegypti* from tyres and could also be used for other species, notably *Ae. albopictus*. The benefits of this are three-fold. First, and most importantly, suspected eggs on the tape can be visualized with a hand-held lens which means fast screening of cargo can be achieved. This could result in a shipment being held or tracked to the onward destination where targeted control methods could be deployed if eggs are confirmed to be from AIMs. We have provided a flow chart to describe how the procedural process for such screening processes could take place (Fig. 5). Additionally, this method could be used to survey any location where tyre sampling is required (i.e. tyre yards, waste piles). Secondly, the ability to hatch and rear eggs through larvae to adults is possible although further field studies will be required to understand how environmental variables could affects factors such as egg mortality. Larval and adult rearing allows for morphological identification and additional downstream investigations (e.g. screening for insecticide susceptibility and transovarial transmission of diseases) [74]. Insecticide resistance screening is important given the worldwide spread of insecticide resistance and because AIMs can be imported from any country where they are present, irrespective of disease presence or absence. Lastly, the tapes tested showed no inhibition of PCR amplification, therefore, additional information can be gained from the DNA of any samples collected (e.g. population genetics). Due to the low cost and potentially high levels of efficiency, further development of this method could allow it to be deployed internationally, acting as an early warning system for new introductions. However, additional validation of this technique in the field would be advantageous to quantify the effects of sampling soiled tyres. We are currently devising a convenient method of applying tape to the tyre surface to produce a standardised methodology.

## Data Availability

All data generated or analysed during this study are included in this published article.

## Abbreviations

AIM(s): *Aedes* invasive mosquito(es)
SEM: scanning electron microscopy
DENV: dengue fever
CHIKV: chikungunya virus
ZIKV: Zika virus
SC: serosal cuticle
PCR: polymerase chain reaction
DT: duct tape
BT: body tape
FT: double sided floor tape
PT: clear packing tape
PHA: port health authorities
